# Antigen-Specific Cytokine and Chemokine Gene Expression for Diagnosing Latent and Active Tuberculosis

**DOI:** 10.3390/diagnostics10090716

**Published:** 2020-09-18

**Authors:** Workneh Korma, Adane Mihret, Yunhee Chang, Azeb Tarekegn, Metasebiya Tegegn, Adem Tuha, Dasom Hwang, Mesfin Asefa, Mahlet O. Hasen, Seoyoung Kim, Tesfaye S. Tessema, Hyeyoung Lee

**Affiliations:** 1 Molecular Diagnostic Laboratory, Department of Biomedical Laboratory Sciences, Yonsei University, Wonju 26493, Korea; jyhskg@yonsei.ac.kr (Y.C.); 2016314391@yonsei.ac.kr (D.H.); tjdyd90@naver.com (S.K.); 2 Institute of Biotechnology, Addis Ababa University, Addis Ababa, P.O. Box 1176, Ethiopia; tesfaye.sisayt@aau.edu.et; 3 Armauer Hansen Research Institute, Addis Ababa, P.O Box 1005, Ethiopia; amihret@gmail.com (A.M.); azititar@gmail.com (A.T.); metii.teg@gmail.com (M.T.); ademyesuf2044@gmail.com (A.T.); mahi2b4@yahoo.com (M.O.H.); 4 St. Paul’s Hospital Millennium Medical College, Department of pathology, Addis Ababa, P.O. Box 1271, Ethiopia; mesfin.asefa@sphmmc.edu.et

**Keywords:** Tuberculosis diagnosis, cytokine and chemokine biomarker, extrapulmonary tuberculosis, gene expression, RT-PCR, diagnostics, tuberculosis biomarker, cytokines, chemokines

## Abstract

Tuberculosis infection exhibits different forms, namely, pulmonary, extrapulmonary, and latent. Here, diagnostic markers based on the gene expression of cytokines and chemokines for differentiating between tuberculosis infection state(s) were identified. Gene expression of seven cytokines (Interferon gamma (IFN-γ), Interferon gamma-induced protein 10 (IP-10), Interleukin-2 receptor (IL-2R), C-X-C Motif Chemokine Ligand 9 (CXCL-9), Interleukin 10 (IL-10), Interleukin 4 (IL-4), and Tumor Necrosis Factor alpha (TNF-α)) in response to tuberculosis antigen was analyzed using real-time polymerase reaction. The sensitivity and specificity of relative quantification (2^^-ΔΔCt^) of mRNA expression were analyzed by constructing receiver operating characteristic curves and measuring the area under the curve (AUC) values. Combinations of cytokines were analyzed using the R statistical software package. IFN-γ, IP-10, IL2R, and CXCL-9 showed high expression in latent and active tuberculosis patients (*p* = 0.001), with a decrease in IL10 expression, and no statistical difference in IL-4 levels among all the groups (*p* = 0.999). IL-10 differentiated pulmonary tuberculosis patients from latent cases with an AUC of 0.731. IL10 combined with CXCL-9 distinguished pulmonary tuberculosis patients from extrapulmonary cases with a sensitivity, specificity, and accuracy of 85.7%, 73.9%, and 81.0%, respectively. IL-10 together with IP-10 and IL-4 differentiated pulmonary tuberculosis from latent cases with a sensitivity and specificity of 77.1% and 88.1%, respectively. Decision tree analysis demonstrated that IFN-γ IL-2R, and IL-4 can diagnose tuberculosis infection with a sensitivity, specificity, and accuracy of 89.7%, 96.1%, and 92.7%, respectively. A combination of gene expression of cytokines and chemokines might serve as an effective marker to differentiate tuberculosis infection state(s).

## 1. Introduction

Tuberculosis is one of the leading causes of morbidity and mortality worldwide that accounted for the death of 1.2 million individuals and demonstrated an incidence of 10 million cases in the year 2018. Ethiopia is one of the thirty high burden countries worldwide with a total incidence rate of 151 and mortality rate of 24 per 100,000 population [1].

It is estimated that one-third of the global (2 billion) population has been infected with *Mycobacterium tuberculosis* (MTB), but only a small proportion (5–10%) will develop an active infection during the following months after the initial infection extending up to the lifetime, whereas the majority of the population demonstrate a latent infection [2]. Consequently, latent tuberculosis infection (LTBI) is believed to be the source of active infection. The state of latency is established due to the host immune response that controls the growth of MTB. Owing to this, the patient remains asymptomatic [3,4].

Improvement in the diagnostic tools is essential for accelerating the progress toward the elimination of tuberculosis as a public health problem by 2050. Mycobacterial culture, the gold standard method of diagnosis of active tuberculosis infection, is a time-consuming process (3 to 6 weeks). Hence, the diagnosis of MTB is primarily performed by sputum smear microscopy; however, it has a low sensitivity ranging from 20 to 60% [5]. The implementation of a rapid molecular test, such as GeneXpert^®^, has improved the detection of active tuberculosis infection with increased sensitivity. However, the diagnosis of extrapulmonary tuberculosis (EPTB) remains challenging due to the nature of the disease, which primarily relies on clinical symptoms and invasive procedures [6].

Interferon gamma (IFN-γ), the primary cytokine produced by host immune cells in response to MTB, is widely utilized to detect tuberculosis infection. [7]. In vitro measurements of the level of IFN-γ in blood against MTB antigens are utilized as a marker in tuberculosis infection. QuantiFERON Gold in tube (QFT-GIT) test, which is an enzyme-linked immunosorbent assay (ELISA) performed with whole blood samples, and enzyme-linked immunospot assay (T-SPOT) test, which is based on peripheral blood mononuclear cells (PBMCs), are the two gamma release assays (IGRAs) commercially available. Both methods utilize the expression of IFN-γ initiated by cluster of differentiation 4 (CD4) lymphocytes in response to ex-vivo stimulation with Region of Difference 1 (RD1)-encoded MTB-specific antigens, namely, culture filtrate protein (CFP-10) and early secretory antigenic target (ESAT-6) [8,9]. Although IGRAs have overcome the drawback of false positivity of the tuberculin sensitivity test (TST) owing to cross-reactivity with the BCG vaccine, it exhibits an inherent failure in discriminating between LTBI and active tuberculosis infection [10]. Therefore, the expression of various cytokines was studied and suggested as potential diagnostic markers that could differentiate various tuberculosis infection statuses. IFN-γ combined with IFN-γ-inducible protein 10 (IP-10) demonstrated an increased sensitivity for the detection of MTB infection [11]. In other studies, mitogen-induced IP-10 response showed higher sensitivity and specificity in distinguishing active tuberculosis and LTBI [12], and IP-10 combined with monocyte chemotactic protein-2 (MCP-2) was also demonstrated to be an effective marker for the diagnosis of tuberculosis infection [11]. QuantiFERON-TB Gold In-Tube (QFT-GIT) test also has a limitation in diagnosing EPTB. Although studies have proposed various diagnostic markers, accurate detection of EPTB remains elusive [13,14]. A previous study has shown that the implementation of combined markers might be useful for distinguishing various infection statuses [15].

MTB interacts initially with alveolar macrophages via cell surface receptors. Subsequently, cascades of signals lead to immune responses. The expression of cytokines is the primary immune response mediated by T-lymphocytes. T cells with CD4 surface molecules are known as T helper cells (Th1 and Th2) [16]. We hypothesized that the gene expression of Th1, Th2, and IFN- γ-induced chemokines might be utilized for the diagnosis of tuberculosis infection. Therefore, Th1 (pro-inflammatory) cytokines, such as IFN-γ, IL-2, and TNF-α; Th2 (anti-inflammatory) cytokines, including IL-4 and IL-10; and IFN-γ-induced chemokines, such as CXCL-9 and CXCL-10, were selected to investigate the gene expression in blood cells stimulated with MTB antigens and compare it with that of unstimulated control blood cells obtained from individuals categorized under four different groups of participants (healthy, latently infected, and smear-positive pulmonary tuberculosis (PTB) and EPTB).

## 2. Materials and Methods

### 2.1. Ethical Statement

The study protocol was reviewed and approved by the National Research Ethics Review Committee (NRERC), No. 3.10/13/2018, Ministry of Innovation and Technology, Ethiopia, and Yonsei University, Wonju Institutional Review Board (IRB) No. 1041849-201709-BR-104-02, South Korea. All participants were informed about the study and provided consent.

### 2.2. Study Participants and Case Definitions

A total of 151 participants (51 healthy controls, 42 latently infected individuals (LTBI), 35 Pulmonary tuberculosis (PTB), and 23 tuberculosis lymphadenitis (TBLN) were recruited during the study period (January 2018 to January 2019) from three hospitals and four health centers in Addis Ababa, Ethiopia. The case definition of participants was based on the WHO classification [17]. PTB patients were clinically diagnosed as new cases and confirmed by sputum smear examinations for acid-fast bacilli (AFB) and/or GeneXpert^®^ and culture. Clinical suspects of lymphadenitis diagnosed with histological examination and culture-confirmed fine needle aspirates (FNA) were categorized under Extra pulmonary tuberculosis (EPTB). None of the latent infected and healthy control individuals had a history of tuberculosis or recent infections, and they were classified based on the clinical evidence and QFT-ELISA assays (Table 1). Household contacts who were QFT-positive were classified as having LTBI, whereas those with negative QFT results were considered healthy controls. Individuals with HIV infection, diabetes, allergic diseases, respiratory infections, and other immunocompromised conditions were excluded from this study.

### 2.3. Interferon Gamma Release Assay (IGRA)

IGRA was performed using the QuantiFERON^®^-TB Gold In-Tube test aided with ELISA. A total of 5 mL of whole blood was collected in heparin-coated tubes (BD Vacutainer^®^, Franklin Lakes, NJ, USA). Then, 1 mL of blood was added to three tubes: nil (control), MTB antigen-coated with (Early Secretary Antigen-6 (ESAT-6), Culture Filtrate Protein-10 (CFP-10), and TB7.7), and mitogen (to be used as a positive control), followed by incubation for 20 h at 37 °C. After incubation, plasma was collected via centrifugation and the levels of IFN-γ were measured by ELISA using the QFT system as per the manufacturer’s protocol [9]. The cells separated from the plasma were treated with 500 mL of Red Blood Cells (RBC) bone marrow stabilization buffer (Roche diagnostic, Manheim, Germany) and stored at −80 °C for subsequent RNA extraction.

### 2.4. RNA Extraction and Complementary DNA (cDNA) Synthesis

Whole blood cells mixed with stabilization buffer and stored under −80 °C were thawed. Total RNA was extracted using the High Pure RNA Isolation Kit (Roche diagnostic, Basel, Switzerland) following the manufacturer’s instructions (https://lifescience.roche.com/global_en/products/high-pure-rna-isolation-kit.html). The quality and concentration of RNA were measured using the Infinite^®^ 200 PRO NanoQuant instrument. Prior to quantitative polymerase reaction, complementary DNA (cDNA) was prepared using the Moloney Murine Leukemia Virus (MMLV)-based reverse transcriptase enzyme, which is a two-step reaction involving the incubation of RNA templates with a master mix at 65 °C and subsequent amplification. Initially, 10 µL of the RNA template was added to a mixture containing 1 µL of 0.25 µg/µL random primer (Invitrogen^®^, Carlsbad, CA, USA) and 4 µL of 2.5 mM deoxynucleotide triphosphates (dNTPs) (iNtRON Biotechnology^®^, Gyeonggi-do, South Korea) and heated at 65 °C to denature the secondary structure of RNA. The reaction was immediately cooled on ice to let the primer anneal to the RNA. Then, 1 µL of the enzyme, MMLV-reverse transcriptase, 2 µL of 0.1 M dithiothreitol (DTT) (Invitrogen^®^), and 4 µL of 5x first strand buffer were added to the heated reaction mixture. Finally, amplification (cDNA synthesis) was performed using Veriti 96 well Thermal Cycler (Applied biosystem^®^, Foster City, CA, USA) with a reaction cycle set as 25 °C for 10 min, 37 °C for 50 min for RT extension, 70 °C for 15 min to inactivate the enzyme, and finally cooling at 4 °C. The synthesized cDNA was stored at −20 °C for real-time polymerase chain reaction (RT-PCR).

### 2.5. Primer, Probe Designing and Optimization

Primers and probes targeting the seven selected genes (IFN-γ, TNF-α, IL-10, IL-4, IL-2R, IP-10, and CXCL-9) were designed using a web application developed by Integrated DNA Technologies, Inc. (https://sg.idtdna.com/Primerquest/Home/Index). The sequences of each gene were entered into the website to generate the primers in line with their exon-exon junctions. The primers and probes were selected based on the parameters described [18] and evaluated in a previous study [19] (Supplementary Material Table S1).

### 2.6. Real-Time Polymerase Chain Reaction Analysis

RT-PCR was performed using the CFX96 Touch Real-Time PCR Detection System (Bio-Rad Laboratories, Inc, Hercules, CA, USA) and the TaqMan^®^ probe that utilizes fluorescent emissions. Sense and antisense primers of IFN-γ, TNF-α, IL-10, IL4, IL2R, IP-10, and CXCL-9 were used to amplify the cDNA, and glyceraldehyde-3 phosphate (GAPDH) was used as a “housekeeping” gene [20]. The RT-PCR master mix containing 10 µL of THUNDERBIRD Probe qPCR Mix (TOOBO, Osaka, Japan), 5 µL of ultra-distilled water, 3 µL mixture of forward and reverse primer, and probe (1 µL of 10 pmol from each), was dispensed in to the PCR tubes. Then, 2 µL of cDNA was added to bring the final reaction volume up to 20 µL. The reaction cycle for RT-PCR was set as follows: 20 s at 95 °C followed by 40 cycles of 3 s at 95 °C and 30 s at 60 °C.

### 2.7. Normality of Sample Distribution

The gene expression results were entered in an excel data sheet following the protocol described in a previous study [21]. The changes in the expression of the gene encoding the MTB antigen between stimulated and non-simulated controls were normalized using the reference gene (GAPDH) expression results. The relative gene expression was calculated using the 2^−ΔΔCT^ method (Supplementary Material Table S2) [21]. The sample distribution was assessed with the normality test using the SPSS software package. The data showed that the distribution was not normal with a significance value of 0.000. Thus, we used the Kruskal-Wallis test and Student’s t-test (Supplementary Material Table S3).

### 2.8. Data Analysis

The expression of mRNA extracted from MTB antigen-stimulated (ESAT-6, CFP-10, and TB7.7) and unstimulated whole blood cells were analyzed by CFX Manager software Version: 3.1. (Bio-Rad Laboratories, Inc.). The threshold (Ct) for each was set to analyze the absolute expression, which is the result of differences between stimulated and non-stimulated expression after normalizing with the expression of the endogenous gene (Supplementary Material Table S4). Then, the relative quantification of the expression of the seven selected target genes was then analyzed using the 2^−ΔΔCT^ method as described in a previous study [21].

The differences in gene expression between two groups were compared using the Mann-Whitney test, whereas the variances among four groups (healthy controls (HC), Latent tuberculosis (LTB), Pulmonary tuberculosis (PTB), and Extra pulmonary tuberculosis (EPTB)) were compared using the nonparametric ANOVA (Kruskal–Wallis) test, and multiple comparisons were corrected with the Dunn’s post-test. The diagnostic performance of cytokines was estimated using the receiver operating characteristic (ROC) curve analysis and calculating the area under the curve (AUC) values. The *p*-value < 0.05 was considered statistically significant. In addition, the diagnostic value of a combination of genes was evaluated by R 3.6.3 foundation statistical computing by processing four types of classifier models (decision tree, logistic regression, neural network, and support vector machines).

## 3. Results

### 3.1. Participants Characteristics

**Figure 1:**
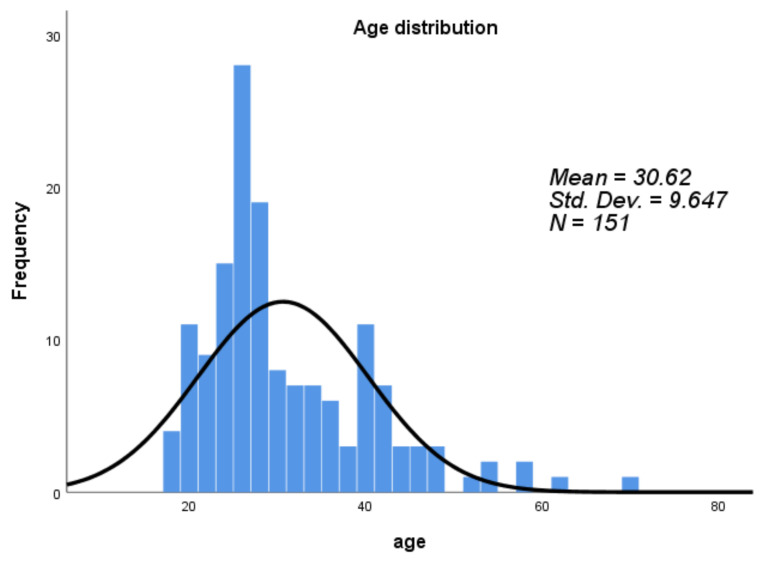
Age distribution of study participants and frequency classified with a gap of 10 years. The mean age was 36.62 ± 9.65 years.

A total of 151 subjects were enrolled, consisting of 51 healthy controls, 42 latently infected patients, 35 PTB patients, and 23 EPTB (TBLN) patients. The number of male participants was higher than that of females, with a male to female ratio of 1.7 (95 males and 56 females). The mean age of participants was 30.62 ± 9.6 years, ranging from 18 to 70 years (Table 1 and Figure 1).

### 3.2. Determination of Cut off Values and Diagnostic Performance of Cytokines and Chemokines

The cutoff values were determined by considering both EPTB and PTB cases as positive, whereas the healthy groups were considered controls, based on the ROC curve analysis. The cut off values for IFN-γ, TNF-α, IL-10, IL2R, IP-10, and CXCL-9 were >1.07, >1.52, <0.55, >2.02, >1.22, and >1.22, respectively. The diagnostic potential of the markers was evaluated by determining the positivity using the relative expression results of each marker. For differentiating PTB, IFN-γ and CXCL-9 exhibited the highest positivity (85.72%) followed by IP-10 and IL-10 with 80%, IL-2R with 74.3%, and TNF-α, which showed the lowest positivity at 62.9% (Supplementary Material Table S4).

### 3.3. Individual Biomarkers Identification and Validation

**Figure 2:**
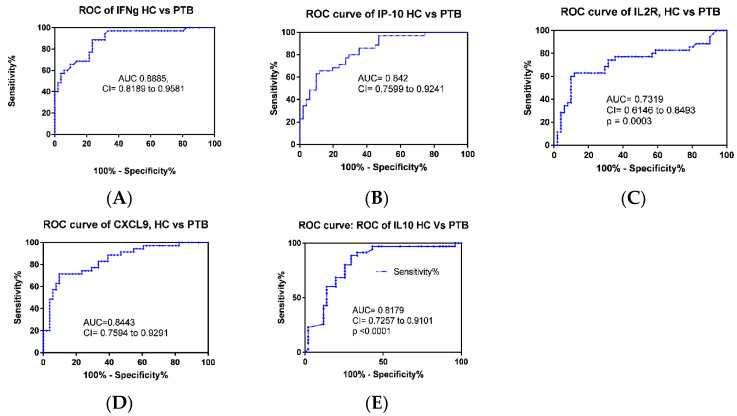
(**A**–**E**) ROC curve analysis of cytokines and chemokines in pulmonary tuberculosis patients (PTB) compared to healthy controls (HC). The genetic expression of Interferon gamma, Interferon gamma-induced protein 10 (IP-10), Interleukin-2 receptor (IL-2R), and C-X-C Motif Chemokine Ligand 9, Interleukin 10 (IL-10) Pulmonary tuberculosis infected patients (*n* = 35), compared with healthy controls (*n* = 51) from A to E respectively.

**Figure 3:**
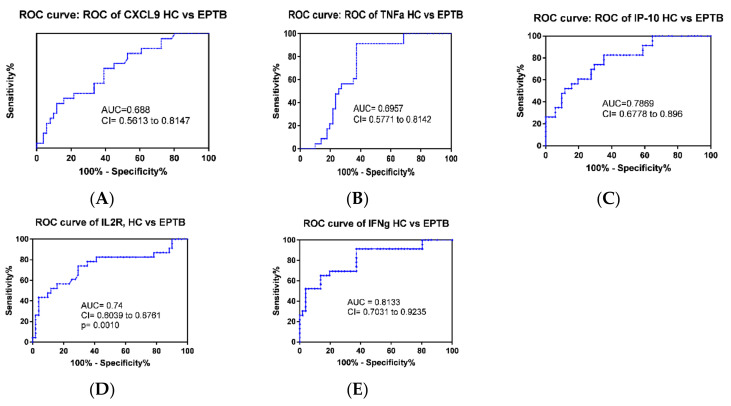
(**A**–**E**) The Receiver Operating Characteristic (ROC) curve analysis of cytokines and chemokines gene expressions of extrapulmonary tuberculosis patients (EPTB), *n* = 23 compared to healthy controls *n* = 51, where the Area Under the Curve (AUC) values of CXCL_9, TNF-α, IP-10, IL-2R, and IFN-γ from A to E respectively.

Multiple comparisons were performed using the Mann–Whitney test and ANOVA to assess the difference in the median values, and ROC curve analysis was also applied to evaluate the discriminatory ability of individual cytokines between different groups (Table 2). Accordingly, four cytokine genes, IFN-γ, IP-10, IL2R, CXCL-9, and TNF-α showed significant difference (*p* = 0.0001) between patients with LTBI and healthy individuals with AUC values of 0.86, 0.85, 0.82, 0.79, and 0.694, respectively. However, the mean value of IL10 and IL4 did not show a significant difference between the two groups with *p* = 0.99 (Table 2). In a similar analysis, five cytokines genes, IFN-γ, IP-10, IL2R, CXCL-9, and IL-10 showed a significant difference (*p* = 0.0001 for all but IL-2R 0.0002) between PTB patients and healthy participants with AUC of 0.89, 0.84, 0.73, 0.84, 0.82, respectively (Figure 2 and Table 2). In the case of EPTB, we observed that the gene expression of five cytokines, namely, IFN-γ, IP-10, IL2R, CXCL-9, and TNF-α, significantly differentiated from healthy controls with *p*-values of 0.0001, 0.0001, 0.0008, 0.009, and 0.007 and AUC values of 0.81, 0.79, 0.74, 0.69, and 0.70, respectively (Figure 3 and Table 2).

**Figure 4:**
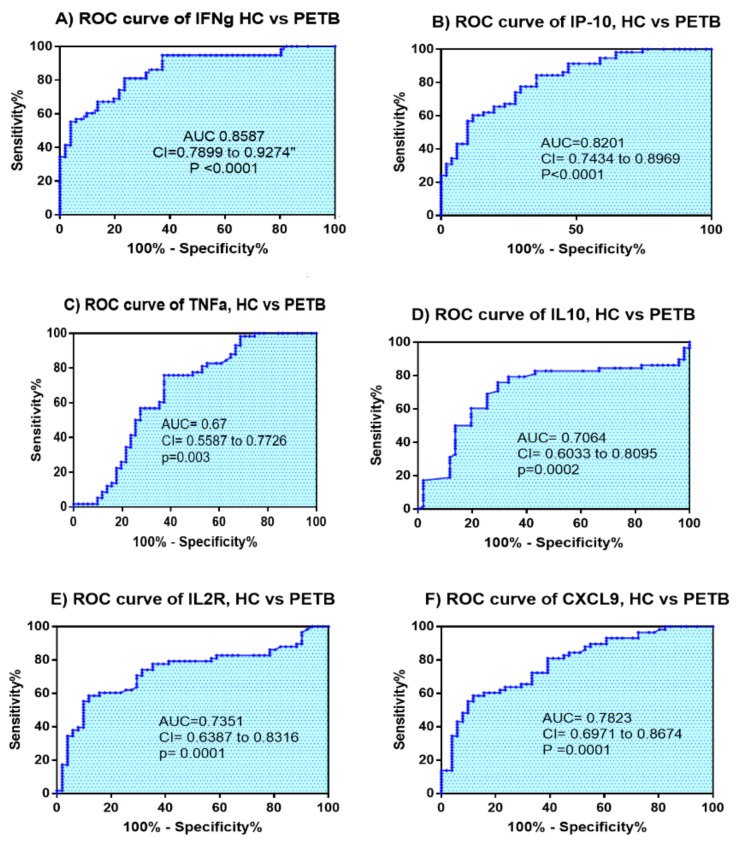
The Receiver Operating Characteristic (ROC) curve analysis of cytokines and chemokines (IFN-γ: (**A**), IP-10: (**B**), TNF-α: (**C**), IL-10: (**D**), IL-2R: (**E**), and CXCL-9: (**F**)) gene expressions of active tuberculosis patients (*n* = 58) compared with healthy controls (*n* = 51). Active tuberculosis infected patient category is the sum of pulmonary and Extrapulmonary infected participants which is represented as PETB. The *p* values (*p* < 0.05) considered as statistically significant.

Moreover, the total tuberculosis patients included the combination of both PTB and EPTB patients. The Mann–Whitney test and multiple comparison ANOVA (Kruskal–Wallis test) demonstrated that six markers, namely, IFN-γ, TNF-α, IP-10, IL-10, IL-2R, and CXCL-9, showed significant differences as compared to healthy controls (Table 2). The *p*-value of four cytokines (IFN-γ, IP-10, IL-2R, and CXCL-9) was 0.0001, whereas the *p*-value of IL-10 and TNF-α was 0.0002 and 0.003, respectively. To analyze the diagnostic potential of these markers in tuberculosis infection, ROC curves were also evaluated. IFN-γ demonstrated the highest AUC value of 0.86, with a 95% confidence interval (CI) ranging from 0.79 to 0.93, followed by IP-10 with AUC = 0.82 and CI ranging from 0.74–0.90, CXCL-9 with AUC = 0.78 and CI ranging from 0.70–0.87, and IL2R with AUC = 0.74 and CI ranging from 0.64–0.832 (Figure 4A–F).

**Figure 5:**
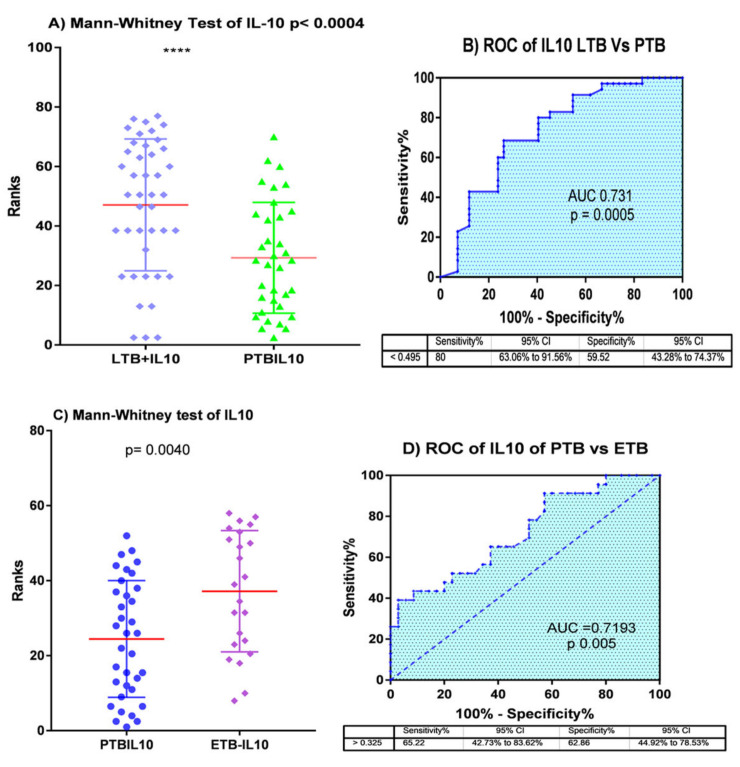
(**A**) Mann-Whitney test result of IL-10 levels between LTBI and PTB patients (**** *p* < 0.0004). (**B**) ROC curve analysis result of IL-10 levels between LTBI and PTB patients. (**C**) Mann-Whitney test result of IL-10 levels between PTB and EPTB patients where the red line shows the median of each groups. (**D**) ROC curve analysis result of IL-10 levels between PTB and EPTB patients.

**Figure 6:**
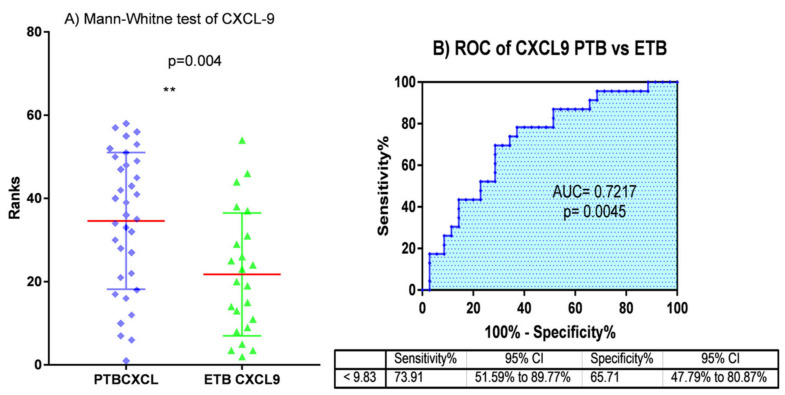
(**A**) Mann-Whitney test (** *p* < 0.004, the red line shows the median of each groups) and (**B**) ROC curve analysis results of CXCL-9 levels between PTB and EPTB patients.

The comparative analysis between PTB and latently infected patients was performed using a non-parametric t-test, the results of which demonstrated that out of the seven genes, IL-10 was the only gene that was able to differentiate LTB from PTB with a *p*-value of 0.0004. In addition, ROC curve analysis was performed to assess the diagnostic potential of IL-10, which showed the AUC value of 0.731 (Figure 5A,B). The Mann–Whitney t-test analysis between PTB and EPTB also demonstrated that both IL-10 and CXCL-9 demonstrated significant differences with *p*-values of 0.004 (Table 2B). Moreover, the evaluation of the diagnostic potential of the two markers (IL10 and CXCX-9) for distinguishing between PTB and EPTB resulted in AUC values of 0.731 and 0.72, sensitivity of 66.2% and 73.9%, and specificity of 62.9% and 65.7%, respectively, in addition to a *p*-value of 0.004 (Figure 5C,D and Figure 6A,B).

**Figure 7:**
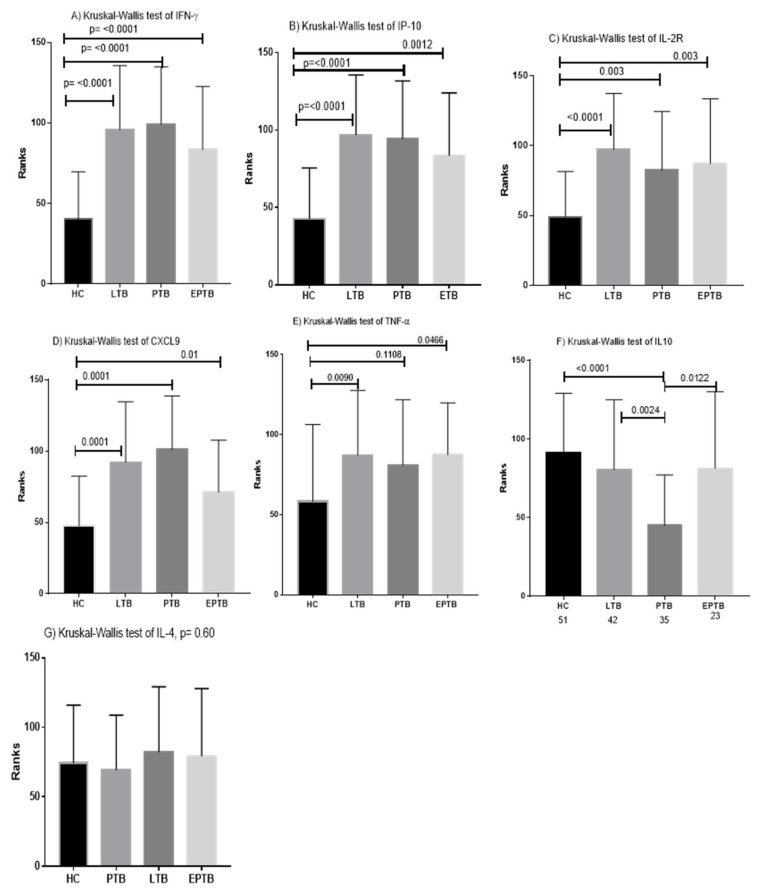
Kruskal–Wallis test of the relative expression of genes among four groups of participants (healthy controls, PTB, LTB and EPTB). The expression value of each groups was different, and the results were presented by comparing each tuberculosis infected groups versus with health controls. Of the seven cytokines, five such as (**A**) IFN-γ, (**B**) IP-10, (**C**) IL-2R (**D**) CXCL-9, and (**E**) TNF-α were shown significant difference between tuberculosis infected (LTB, PTB and EPTB) and healthy controls with the *p* values written on each figure whereas IL-4 (**G**) showed no statistically significant differences. IL10 (**F**) showed significance differences between PTB and LTB or PTB and HC but no differences between LTB and HC.

In general, the analysis of variations in the relative gene expression among four groups of participants (HC, LTB, PTB, and EPTB) using Kruskal-Wallis and Mann-Whitney tests demonstrated that the expression of five genes, namely, IFN-γ, IP-10, IL2R, CXCL-9, and TNFα was increased significantly in the LTBI, PTB, and EPTB cases compared to healthy controls with varied *p*-values, whereas no significant differences were observed in IL4 expression (*p* = 0.6023). The level of IL10 was lower in PTB than in other groups (Figure 7). In addition, four genes, namely, IFN-γ, IP-10, IL2R, and CXCL-9, were strongly expressed and demonstrated a high diagnostic potential for tuberculosis (Table 2).

### 3.4. Combination of Cytokine Markers in Discriminating Tuberculosis Infection 

The diagnostic efficiency of the combinations of genes was assessed with the R software. The qPCR data stored in excel were entered into a program designed to run automatically with four models using R script: decision tree, logistic regression, neural network, and support vector machine. The training, validation, and test sets were assigned with a ratio of 8:2:2, respectively. The R program was run with 4000 iterations. The convergence result showed that a combination of cytokines could differentiate between different tuberculosis state(s) with better sensitivity, specificity, and accuracy. Accordingly, the data obtained from four groups of participants were analyzed in six different comparison setups (Table 3 and Supplementary Material Table S5).

**Figure 8:**
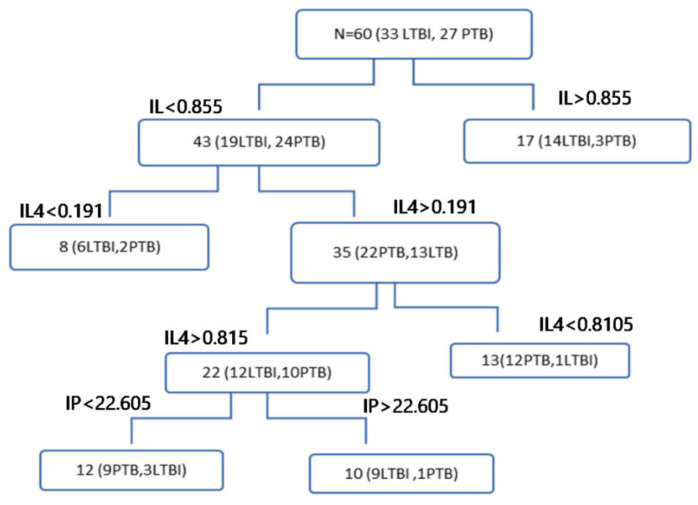
Machine learning-based decision tree comparing PTB and LTBI cases. The combination of IL-10, IL-4, and IP-10 distinguishes PTB from LTBI with a sensitivity, specificity, and accuracy of 77.1%, 88.1%, and 83.1%, respectively.

The decision tree analysis showed that IL 10, IL 4, and IP 10 were able to differentiate between LTBI and active tuberculosis with 77.1% sensitivity, 83.1% specificity, and 88.1% accuracy (Table 3 and Figure 8). Moreover, a combination of IFN-γ, IL-10, and IL-2R was able to differentiate PTB from healthy controls with a test result of 97.1% sensitivity 88.2% specificity, and 91.9% accuracy. In addition, IFN-γ, IL-2R, and IL4 were identified as effective markers to differentiate both EPTB and PTB from healthy controls with a sensitivity of 89.7%, specificity of 96.1%, and accuracy of 96.1%. IL-10 combined with CXCL-9 could also differentiate EPTB from PTB with 85.7% sensitivity, 73.9% specificity, and 81.0% accuracy (Table 3). In a similar analysis, latently infected plus healthy control groups were compared with actively infected participants (both pulmonary and extra pulmonary tuberculosis), pulmonary tuberculosis and extrapulmonary tuberculosis, respectively (Supplementary Material Table S5).

## 4. Discussion

A QuantiFERON^®^-TB Gold In-Tube assay that measures the level of IFN-γ released in response to MTB antigens was utilized to detect tuberculosis infection. However, it cannot differentiate LTBI from active tuberculosis infection cases [10]. Consequently, the development of a rapid and noninvasive bioassay is essential for the diagnosis of tuberculosis infection status. Different studies have proposed that both individual and/or combined cytokines can act as potential biomarkers in differentiating PTB from LTBI [12,15,19,22,23] as well as for the detection of EPTB [13,14,24]. In this study, we employed RT-qPCR to analyze the relative gene expression of cytokines that were stimulated as a part of the bioassay for discriminating tuberculosis infection status, which offers a better advantage with increased sensitivity and specificity. Thus, the genetic expression of selected cytokines was used in differentiating the tuberculosis infection state of participants categorized in four groups: PTB, EPTB (TBLN), latently infected (QFT-positive), and healthy controls (QFT-negative) (Table 2 and Table 3).

We observed that, of the seven cytokine genes analyzed by non-parametric t-tests, the expression of five genes, namely, IP-10, CXCL-9, IFN-γ, IL-2R and IL-10, was significantly different in PTB compared to the healthy controls with AUC values of 0.842, 0.844, 0.889, 0.732, and 0.818, respectively (Figure 2). In accordance with our results, the expression of cytokines, namely, IFN-γ, IL-2R, and IP-10 was reported to be significantly higher in tuberculosis patients than in healthy individuals in a previous study [19]. In another study, IFN-γ, IL-4, IP-10, and TNF levels in plasma were able to discriminate tuberculosis patients from household contacts with high sensitivity. In contrast, none of them were competent to differentiate between QFT-positive and QFT-negative household contacts [25]. 

The response of the hosts to mycobacterial infection involves a wide range of cytokines and chemokines at different levels of the immune system. Various studies have proposed a multiplex model of cytokines and chemokines as biomarkers to distinguish tuberculosis infection status owing to the advantages of increased sensitivity and specificity over the use of single cytokines and/or chemokines [19,26]. Hence, combined detection of IFN-γ, IP-10, and Monokine-induced-by-IFN-γ (MIG) exhibits better diagnostic performance for tuberculosis than the individual cytokine/chemokine assays [26]. Cytokines, such as IFN-γ, IL-1RA, IL-8, and MCP-1, were also differentially expressed at higher levels in tuberculosis patients compared to latently infected participants. IL10 and IL-5 levels were lower in latently infected individuals than tuberculosis patients [22]. In contrast, our study showed that the levels of IL10 were lower in PTB patients than in any other form of tuberculosis (Figure 7). In this study, we utilized both combined and individual cytokine analysis to discriminate PTB from latently infected participants and healthy controls. Accordingly, IL-10 was a single cytokine that differentiated PTB from latently infected participants with a *p*-value of 0.0005 and AUC 0.731 (Figure 5A,B), which is in contrast to the previous study wherein IL10 levels were higher in in PTB patients than in latently infected individuals [22]. In parallel, the diagnostic capacity of combined cytokines using decision tree analysis demonstrated that the combination of IL10, IL4, and IP10 could distinguish PTB patients from latently infected individuals with a sensitivity, specificity, and accuracy of 77.1%, 88.1%, and 83.1%, respectively (Table 3). Similarly, a previous study also reported that IL10 and IL2 accurately identified active tuberculosis from LTBI cases [23]. Moreover, the expression of combinations of Th1 type factors (TNF-α and IL-2R) and IFN-γ-induced chemokines (CXCL9 and CXCL10) potentially differentiated PTB from latently infected participants and healthy controls and identified the combinations of IFN-γ, TNFα, and IL-2R and TNF-α, IL-2R, CXCL-9, and IP-10 as the best diagnostic markers for active and LTBI, respectively [19]. 

Accurate diagnosis of EPTB remains obscure. Goyal et al., suggested that the serum level of circulating IFN-γ and the ratio of IFN-γ and IL-2 remarkably identified EPTB patients from healthy controls [13]. Our study showed that, of the seven markers analyzed IFN-γ, IP10, IL2R, TNF-α and CXCL9 distinguished pulmonary tuberculosis cases from health controls with AUC value of 0.813, 0.79, 0.74, 0.7 and 0.69 respectively(Figure 3).In another study, the plasma level of VEGF was demonstrated to be a biomarker for differentiating EPTB from non-extrapulmonary forms [24]. In this study, individual cytokine analysis using a non-parametric t-test and ROC curve revealed that IL10 and CXCL-9 were able to differentiate EPTB from PTB cases (Figure 5C,D and Figure 6). Similarly, analysis using machine learning also showed that a combination of IL10 and CXCL9 had a diagnostic capacity to identify EPTB from PTB with a sensitivity, specificity, and accuracy of 85.7%, 73.9%, and 81% (Table 3). 

In addition, the analysis of PTB and EPTB as one group (PETB) to identify common markers using both machine learning and non-parametric t-test methods was performed. Accordingly, of the seven genes, the following five cytokines: IFN-γ, IP10, IL10, IL2R, and CXCL9 were identified as markers that individually differentiated PETB cases from healthy controls (Figure 4). In contrast, the decision tree analysis showed that combined gene expression of IFN-γ, IL-2R, and IL4 differentiated tuberculosis patients (both PTB and EPTB) from healthy participants with sensitivity, specificity, and accuracy of 89.7%, 96.1%, and 92.7%, respectively. Hence, IFN-γ and IL-2R are common markers identified and associated with tuberculosis (Table 3).

## 5. Conclusions

The development of rapid and accurate diagnostic markers is important for controlling and treating tuberculosis. Differentiating active tuberculosis cases from latent cases still lacks accurate methods. In this study, we observed that IL10 combined with IL 4 and IP 10 could act as a marker to differentiate PTB from LTBI. In addition, the combination of IL10 with CXCL-9 was demonstrated to be an effective marker for differentiating active pulmonary cases from EPTB (TBLN). Based on the machine learning analysis results, IFN-γ and IL2R were found to be the common markers associated with tuberculosis. Thus, combined cytokine and chemokine markers exhibit better potential for diagnosis and differentiation of tuberculosis infection state(s).

## Figures and Tables

**Table 1:** Categorization of participants by sex, percentile proportion, and QuantiFERON enzyme-linked immunosorbent assay (QFT-ELISA) test results after 20 h of incubation at 37 °C.

		Healthy Control	Latent Tuberculosis	PTB	EPTB
Sex	Male	27	29	26	13
	Female	24	13	9	10
Total		51	42	35	23
	%	33.8	27.8	23.2	15.2
	QFT (+/−)	0/51	42/0	34/1	23/0

**Table 2:** The cytokines and chemokines gene expression results compared among different groups. A/ Multiple comparison result among the groups using Kruskal-Wallis Test (One-way Analysis of Variances (ANOVA)) B comparison between two groups using Mann–Whitney U test. The p value for the statistical analysis were set as 0.05 and 95% CI.

	A Multiple Comparison ANOVA (Kruskal-Wallis Test)					
Marker	HC vs. LTB	HC vs. PTB	HC vs. EPTB	HC vs. PTB, EPTB	LTB vs. PTB	PTB vs. EPTB
INF-γ	0.0001	0.0001	0.0015	0.0001	0.999	0.999
TNF-α	0.0233	0.2582	0.1256	0.039	0.999	0.999
IP-10	0.0001	0.0001	0.0035	0.0001	0.999	0.999
IL-10	0.999	0.0001	0.999	0.0022	0.0065	0.0472
IL-2R	0.999	0.0081	0.006	0.0004	0.999	0.999
IL-4	0.0001	0.999	0.999	0.999	0.999	0.999
CXCL-9	0.0001	0.0001	0.5803	0.0001	0.999	0.1066
	**B Mann-Whitney Test (Two-Sided)**					
Marker	HC vs. LTB	HC vs. PTB	HC vs. EPTB	HC vs. PTB, EPTB	LTB vs. PTB	PTB vs. EPTB
IFN-γ	0.0001	0.0001	0.0001	0.0001	0.7875	0.1436
TNF-α	0.0012	0.0216	0.0068	0.0027	0.5033	0.8514
IP-10	0.0001	0.0001	0.0001	0.0001	0.7104	0.2912
IL-10	0.252	<0.0001	0.6198	0.0002	0.0004	0.0045
IL-2R	0.0001	0.0002	0.0008	<0.0001	0.1222	0.5781
IL-4	0.4008	0.6043	0.6118	0.9265	0.1941	0.4414
CXC-L-9	0.0001	<0.0001	0.0094	<0.0001	0.3839	0.004

**Table 3:** Decision tree analysis results obtained using R. The analysis of cytokine gene expression performed to distinguish different categories was programmed using R-statistical software package with a ratio of 6:2:2 corresponding to validation, training, and test sets, respectively. A total of 151 participants, with 51 healthy controls, 42 latently infected participants, 35 smear-positive PTB patients, and 23 clinically and cytologically confirmed EPTB patients were analyzed.

		Decision Tree Analysis Result							
Category		Control	Patient	Total	Normality	Accuracy	Sensitivity	Specificity	Genes
HC vs. LTB	Train	40	33	73	54.8%	86.3%	75.8%	95.0%	IFN-γ, IL2R
	Validate	11	9	20	55.0%	100.0%	100.0%	100.0%	
	Test	51	42	93	54.8%	89.2%	81.0%	96.1%	
		Control	Patient	Total	Normality	Accuracy	Sensitivity	Specificity	
HC vs. PTB	Train	40	27	67	59.7%	89.6%	96.3%	85.0%	IFN-γ, IL10, IL2R
	Validate	11	8	19	57.9%	100.0%	100.0%	100.0%	
	Test	51	35	86	59.3%	91.9%	97.1%	88.2%	
		Control	Patient	Total	Normality	Accuracy	Sensitivity	Specificity	
LTB vs. PTB	Training	33	27	60	55.0%	83.3%	77.8%	87.9%	IL10, IL4, IP10
	Validation	9	8	17	52.9%	82.4%	75.0%	88.9%	
	Test	42	35	77	54.5%	83.1%	77.1%	88.1%	
		Control	Patient	Total	Normality	Accuracy	Sensitivity	Specificity	
EPTB vs. PTB	Training	18	27	45	40.0%	75.6%	81.5%	66.7%	IL10, CXCL-9
	Validation	5	8	13	38.5%	100.0%	100.0%	100.0%	
	Test	23	35	58	39.7%	81.0%	85.7%	73.9%	
HC vs. PETB		Control	Patient	Total	Normality	Accuracy	Sensitivity	Specificity	
	Training	40	46	86	46.5%	90.7%	87.0%	95.0%	IFN-γ, IL-2R, IL4
	Validation	11	12	23	47.8%	100.0%	100.0%	100.0%	
	Test	51	58	109	46.8%	92.7%	89.7%	96.1%	

## Supplementary Materials

The following are available online at https://www.mdpi.com/2075-4418/10/9/716/s1, Table S1: Primer and probe sequences used for quantification, Table S2: Determination of the relative expression of cytokines using 2^−ΔΔCt^ method, Table S3: Normality test of relative gene expression of cytokines, Table S4: Threshold values, sensitivity, specificity, and cut off values for cytokines and chemokines, Table S5: Decision tree (DT) analysis of combined cytokines gene expression using R program that run with 4000 iterations.
