# Supplementary material for: Antigen-Specific Cytokine and Chemokine Gene Expression for Diagnosing Latent and Active Tuberculosis
Source: Diagnostics (Basel). 2020 Sep 18;10(9):716. doi: 10.3390/diagnostics10090716 (PMC7555064; doi:10.3390/diagnostics10090716)
Supplement: Supplementary file 1 [file diagnostics-10-00716-s001.pdf]

Supplementary Tables

Supplementary Table 1 Primer and probe sequences used for quantification

Target name	Primers and TaqMan probes	Primer and TaqMan probe sequences (5' to 3')
INF-γ	Taq-IFN-γ-2F	TGA ATG TCC AAC GCA AAG CA
	Taq-IFN-γ-2R	CGA CCT CGA AAC AGC ATC TGA
	TaqMan-IFN-γ-3	FAM-CGC CAG CAG CTA AAA CAG GGA AGC G-BHQ1
TNF-α	TNF-α-2F	CTC CCC AAG AAG ACA GGG GG
	TNF-α-2R	GGG AAC TCT TCC CTC TGG GG
	TNF-α-TaqMan	FAM-CAG GCG GTG CTT GTT CCT CAG-BHQ1
IL-2R	IL-2R-4F	TGC AAA GTC CAA TGC AGC CAG
	IL-2R-3R	TTC CCA TGG TGG AGG TTC CC
	IL-2R-TaqMan-4	FAM-FGG ACC AAG CGA GCC TTC CAG GTC A-BHQ1
IL-4	IL-4-3F	CAC AGC AGT TCC ACA GGC ACA
	IL-4-3R	TGG CTT CCT TCA CAG GAC ACC
	IL-4-TaqMan-4	FAM-CCG ATT CCT GAA ACG GCT CGA CAG G-BHQ1
IL-10	IL-10-2F	GGG TTG CCA AGC CTT GTC TG
	IL-10-2R	GAA GAA ATC GAT GAC AGC GCC
	IL-10-TaqMan	FAM-CCC TGG GGG AGA ACC TGA AGA CCC-BHQ1
CXCL-9	CXCL9-F	CTA ATT CTT GGG TGT TTA TCC TAT C
	CXCL9-R	ACA GTA TTA TTA GGC ACT GTG GAA G
	CXLX9-TaqMan	FAM-TTG TCA GCT CCT TGA GGG CAA GAG-BHQ1
CXCL-10 (IP-10)	IP-10-2F	CCA GAA TCG AAG GCC ATC AAG A
	IP-10-2R	AGG GAA GTG ATG GGA GAG GCA
	IP-10-TaqMan	FAM-TGC AGT GCT TCC AAG GAT GGA CCA CA-BHQ1

Supplementary Table 2 Determination of the relative expression of cytokines using 2^ -ΔΔCt method. Absolute value from real-time PCR of both TB antigen-stimulated and unstimulated samples with specific markers and the endogenous gene entered an excel sheet. The change in between the control and stimulated was calculated by normalizing with GAPDH, (E and F). ΔΔ Ct (G) denotes the differences between the antigen-stimulated and non-stimulated value of delta Ct. The relative quantification (RQ) Tb antigen-stimulated (J) was finally calculated by 2^-ΔΔCt.

GAPDH Ct value		Gene X CT value		Marker X delta Ct		Marker X ΔΔ Ct		Marker-X RQ Ct	
Nil	TB Ag	Nil	TB-Ag	Nil	TB-Ag	Nil	TB-Ag	Nil	TB-Ag
19.58	19.41	27.81	24.19	8.2	4.8	0.0	(3.4)	1.00	10.91
A	B	C	D	E=C-A	F=D-B	G=E-E	H=F-E	I=2^{G}	J=$2^{\wedge - H}$

Supplementary Table 3 Normality test of relative gene expression of cytokines. The test of normality with sig. a value less than 0.05 implies that the data deviate from normal distribution. Thus, the nonparametric criteria were applied for data analysis using ANOVA and t-test.

Tests of Normality

	Kolmogorov-Smirnov[a]			Shapiro-Wilk		
	Statistic	df	Sig.	Statistic	df	Sig.
IFN-γ	0.300	151	.000	0.539	151	.000
IP-10	0.317	151	.000	0.522	151	.000
TNF-α	0.340	151	.000	0.443	151	.000
CXCL9	0.306	151	.000	0.560	151	.000
IL2R	0.289	151	.000	0.479	151	.000
IL4	0.361	151	.000	0.242	151	.000
IL10	0.431	151	.000	0.141	151	.000

a. Lilliefors Significance Correction

Supplementary Table 4 Threshold values, sensitivity, specificity, and cut off values for cytokines and chemokines. Noninfected healthy individuals were considered controls and compared with tuberculosis patients (both pulmonary and extra-pulmonary).

R.No.	Markers	threshold value (Ct)	Cutoff values	PTB		EPTB	
				Positivity (%)	Negativity (%)	Positivity (%)	Negativity (%)
1	IFN-γ	500	1.07	30(85.52%)	5 (14.2%)	16 (69.6%)	7(30.4%)
2	TNF-α	600	1.52	22(62.9%)	13(37.1)	17 (74%)	6 (26%)
3	IL-10	400	0.55	28(80%)	7(20%)	16 (69.6%)	7(30.4%)
4	IP-10	700	1.22	28(80%)	7(20%)	19 (82.6%)	4 (17.4%)
5	IL-2R	700	2.02	26(74.3%)	9(35.7%)	16 (69.6%)	7(30.4%)
6	CXCL-9	700	1.22	30(85.52%)	5 (14.2%)	15 (65.2%)	8 (34.8%)

Supplementary Table 5 Decision tree (DT) analysis of combined cytokines gene expression using R program that run with 4000 iterations. The expression of healthy controls plus latently infected individuals was compared with actively infected participants (both pulmonary and extrapulmonary tuberculosis), pulmonary tuberculosis and extrapulmonary tuberculosis, respectively. Seven selected cytokines were an input (TNF-α, CXCL9, IFN-γ, IL10, IP10, IL4 and IL2R) for the analysis.

Category	Sample				DT		
HC+LTB Vs PTB + EPTB	Control	Patient	Total	normality	accuracy	sensitivity	specificity
Train	73	146	119	61.3%	80.7%	82.6%	79.5%
Validate	20	12	32	62.5%	62.5%	75.0%	55.0%
Test	93	58	151	61.6%	76.8%	81.0%	74.2%
					IFN-γ, IL.10, IL.2R, CXCL9, IL4		
HC+LTB vs PTB	Sample				DT		
	Control	Patient	Total	normality	accuracy	sensitivity	specificity
Train	73	27	100	73.0%	86.0%	63.0%	94.5%
Validate	20	8	28	71.4%	96.4%	87.5%	100.0%
Test	93	35	128	72.7%	88.3%	68.6%	95.7%
					IFN-γ, IL.10, IL.2R		
HC+LTB vs EPTB	Sample				DT		
	Control	Patient	Total	normality	accuracy	sensitivity	specificity
Train	73	18	91	80.2%	89.0%	66.7%	94.5%
Validate	20	5	25	80.0%	96.0%	80.0%	100.0%
Test	93	23	116	80.2%	90.5%	69.6%	95.7%
					TNF-α, CXCL9, IFN-γ, IL-10		
